# Measuring infectious SARS-CoV-2 in clinical samples reveals a higher viral titer:RNA ratio for Delta and Epsilon vs. Alpha variants

**DOI:** 10.1073/pnas.2116518119

**Published:** 2022-01-20

**Authors:** Hannah W. Despres, Margaret G. Mills, David J. Shirley, Madaline M. Schmidt, Meei-Li Huang, Pavitra Roychoudhury, Keith R. Jerome, Alexander L. Greninger, Emily A. Bruce

**Affiliations:** ^a^Department of Microbiology and Molecular Genetics, Robert Larner, M.D. College of Medicine, University of Vermont, Burlington, VT 05405;; ^b^Virology Division, Department of Laboratory Medicine and Pathology, University of Washington, Seattle, WA 98105;; ^c^Data Science Department, Faraday, Inc., Burlington, VT 05405;; ^d^Vaccine and Infectious Disease Division, Fred Hutchinson Cancer Research Center, Seattle, WA 98109

**Keywords:** SARS-CoV-2, viral load, infectivity, Delta, variant

## Abstract

Novel severe acute respiratory syndrome coronavirus 2 (SARS-CoV-2) variants pose a challenge to controlling the COVID-19 pandemic. Previous studies indicate that clinical samples collected from individuals infected with the Delta variant may contain higher levels of RNA than previous variants, but the relationship between levels of viral RNA and infectious virus for individual variants is unknown. We measured infectious viral titer (using a microfocus-forming assay) and total and subgenomic viral RNA levels (using RT-PCR) in a set of 162 clinical samples containing SARS-CoV-2 Alpha, Delta, and Epsilon variants that were collected in identical swab kits from outpatient test sites and processed soon after collection. We observed a high degree of variation in the relationship between viral titers and RNA levels. Despite this, the overall infectivity differed among the three variants. Both Delta and Epsilon had significantly higher infectivity than Alpha, as measured by the number of infectious units per quantity of viral E gene RNA (5.9- and 3.0-fold increase; *P* < 0.0001, *P* = 0.014, respectively) or subgenomic E RNA (14.3- and 6.9-fold increase; *P* < 0.0001, *P* = 0.004, respectively). In addition to higher viral RNA levels reported for the Delta variant, the infectivity (amount of replication competent virus per viral genome copy) may be increased compared to Alpha. Measuring the relationship between live virus and viral RNA is an important step in assessing the infectivity of novel SARS-CoV-2 variants. An increase in the infectivity for Delta may further explain increased spread, suggesting a need for increased measures to prevent viral transmission.

Despite the rapid development of effective vaccines against severe acute respiratory syndrome coronavirus 2 (SARS-CoV-2), the emergence of novel viral lineages poses challenges to controlling the ongoing COVID-19 pandemic. In the fall of 2020, the Alpha (B.1.1.7) variant emerged in the United Kingdom and was associated with increased transmission and spread (1). By late 2020, the Epsilon (B.1.429/B.1.427) lineage emerged in the US state of California with rapid spread and signs of partial immune evasion (2, 3), before being overtaken by the Alpha variant later that year (4). The Delta (B.1.617.2) variant, first detected in India in early 2021, has outpaced both the Alpha and Epsilon variants and accounts for >95% of global viral sequences (5). Delta’s rapid spread appears to be partially due to increased viral fitness conferred by mutations in the furin cleavage site that increase the efficiency of viral entry (6). Viral RNA levels in samples from people infected with Delta are higher (for a longer duration) than those infected with previous variants, and may be similar in vaccinated and unvaccinated individuals (7–9).

While improved genomic surveillance allows almost real-time detection of viral lineages, functional characterization is required to better understand which mutations underlie increases in viral transmission. One difficulty of interpreting viral infectivity lies in the widespread use of viral RNA levels (measured by RT-PCR in COVID-19–positive clinical samples) as a proxy for viral load. This assumes that there is a relationship between RNA and infectious viral levels, but it is unlikely to be a fixed ratio. The genome to plaque-forming units (PFUs) ratio for SARS-CoV-2 is reported to be 10^3^:1 to 10^6^:1, while SARS is thought to be comparatively more infectious per particle [360:1 (10, 11)]. Quantitative measurement of replication-competent virus in clinical specimens would improve the ability to determine and interpret infectious viral loads for current and future variants.

## Results

**Fig. 1.**
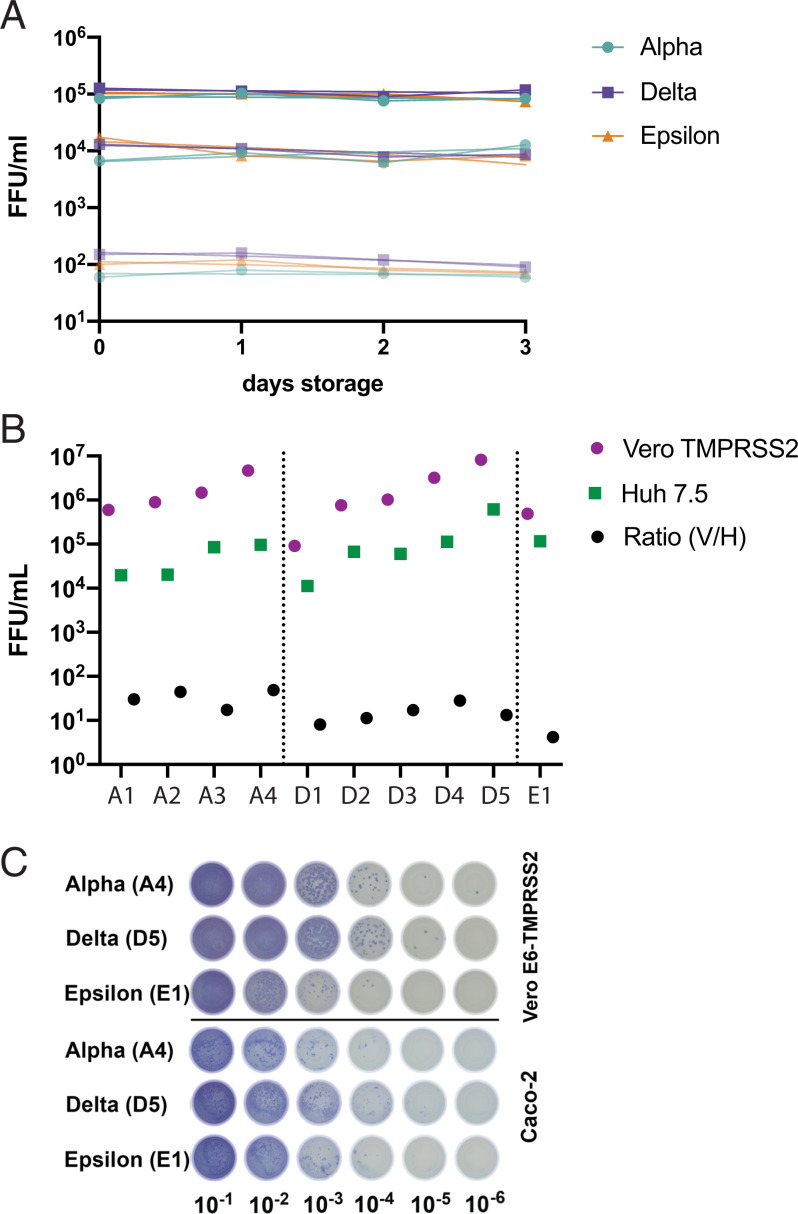
Effect of storage time and cell type on infectious titers. (*A*) Stability of infectious virus from Alpha, Epsilon, or Delta variants was measured by focus-forming assay using viral stocks diluted 1:10, 1:100, or 1:10,000 in Dulbecco’s modified Eagle’s medium and stored at 4 °C for the indicated times. (*B* and *C*) Titers of viral stocks obtained from independent Alpha (A1-A4), Delta (D1-5), and Epsilon (E1) patient specimens were measured in parallel in Vero-TMPRSS2, Huh7.5, and Caco-2 cells by focus-forming assay. (*B*) Individual foci were counted for Vero-TMPRSS2 and Huh7.5 cells; (*C*) overall anti–SARS-CoV-2 N staining is shown, as Caco-2 cells do not form countable foci.

We previously found that infectious virus and RNA from SARS-CoV-2 WA-1 virus stocks are stable at 4 °C (clinical specimens storage temperature) for >4 d (12). To confirm that stability does not differ for variants, we measured viral titers for Alpha, Epsilon, and Delta stocks stored at 4 °C at varying concentrations (Fig. 1*A*). We did not observe any statistically significant decreases in viral stability over 3 d of storage. We also tested stability at 25 °C, 32 °C, and 37 °C and observed no differences between variants (*SI Appendix*, Fig. S1). We performed our focus assay in VeroE6 cells expressing the TMPRSS2 protease which increases the assay sensitivity and allows us to detect low-titer clinical specimens (12). To determine whether the relative infectious titers for each variant differ between human cell lines, we measured a selection of Alpha, Delta, and Epsilon stocks in VeroE6-TMPRSS2, Huh7.5, and Caco-2 cells in parallel (Fig. 1 *B* and *C*). As expected, Vero-TMPRSS2 cells were the most sensitive of the tested cell lines. All three variants showed the same relative titers (quantitatively in Huh7.5 cells, which form discrete foci, and qualitatively in Caco-2 cells, where comparative levels of antigen were observed), and a cell type–variant interaction effect was not observed.

**Fig. 2.**
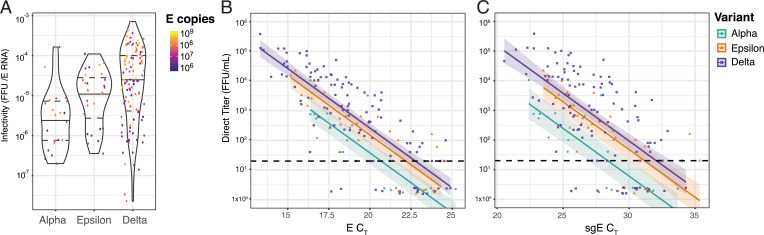
Epsilon and Delta are more infectious than Alpha in clinical specimens. A set of 162 clinical specimens from individuals infected with the Alpha (*n* = 20), Epsilon (*n* = 29), or Delta (*n* = 55 + 58) SARS-CoV-2 variants was used to visualize the relationship between viral titer and viral RNA level for each variant. (*A*) Average infectivity, calculated as the FFU divided by estimated E gene RNA copy number (colored according to the quantity of E gene copies for in the patient specimen). (*B* and *C*) Individual specimen measurements of (*B*) total E RNA (C_T_) and (*C*) subgenomic E RNA on the *x* axis plotted against viral titer (FFU/mL) on the *y* axis. Dashed line indicates the limit of detection for infectious titer (20 FFU/mL). Samples for which we could not measure a viral titer were assigned fixed values of one-tenth the limit of detection, that is, 2 FFU, and randomly assigned a value between 1.5 and 2.5 for display purposes. Lines of best fit and 95% CIs were generated by linear regression on log-transformed data.

To measure the relationship between infectious viral titers of clinical specimens and viral RNA, we performed viral focus assays and RT-qPCR on a set of 162 specimens consisting of Alpha, Epsilon, and Delta variants. These specimens were collected with identical kits from community surveillance outpatient testing sites, and were processed in <54 h. Delta samples had significantly higher (10×) levels of infectious virus, on average, compared to Alpha (*P* = 0.012; Tukey–honest significant difference [HSD] test), while the differences between Alpha and Epsilon or Delta and Epsilon were not statistically different. As the “particle to PFU ratio” is often used to describe viral infectivity, we plotted the “focus forming unit (FFU) to RNA ratio” for each sample in our dataset (Fig. 2*A*). Delta samples had an FFU:RNA ratio of 7.6 times (based on E) and 14.7 times (based on subgenomic E [sgE]) higher than Alpha with a *P* value *P* = 0.0002 and *P* < 0.0001, respectively (Tukey-HSD test). Epsilon samples trended to higher average FFU:RNA ratio than Alpha, with Epsilon 3.7 times (based on E) and 7.1 times (based on sgE) more than Alpha (*P* = 0.78 and 0.017; Tukey-HSD). This trend was also observed in tissue culture, when cells were synchronously infected with stocks of each variant (*SI Appendix*, Fig. S2). High levels of variability in the clinical Delta FFU:RNA ratios may reflect a more heterogeneous vaccination status among the population during the Delta wave compared to earlier Alpha/Epsilon circulation.

We also compared the relationship between FFU and RNA for each variant by generating lines of best fit using linear regression on log-transformed data for total levels of E RNA (Fig. 2*B*) and sgE RNA (Fig. 2*C*). We observed a positive correlation between the amount of viral RNA and infectious virus in individual clinical specimens for all three variants (Dataset S1). Our data suggest that Delta and Epsilon have 6.9 and 4.0 times more infectious virus than Alpha for samples with the same amount of total viral RNA (*P* < 0.0001 and 0.014, respectively). We observed an even stronger trend when comparing infectious virus to sgE RNA levels (15.3×, *P* < 0.0001; 7.9×, *P* = 0.004) (Fig. 2*C*). Subgenomic RNA (sgRNA) has been proposed as a marker of replicating virus, although we and others have seen limited evidence that sgRNAs are exclusively present during viral replication (12). Nonetheless, the sgE data indicate that Delta may have substantially increased (>7 times) infectivity compared to Alpha.

## Discussion

Limitations of this study include lack of access to individual patient-level metadata, which may obscure the effects of age, preexisting conditions, days from exposure/symptom onset, and vaccination status on infectivity. In addition, we examined total RNA (E) and subgenomic (E), but did not have sufficient residual volume to probe genomic RNA (i.e., ORF1a/b). Furthermore, we cannot compare all three variants from samples collected contemporaneously. We have attempted to address this by analyzing only samples that were collected from outpatient community surveillance settings during an upswing in cases, using identical swab kits, to minimize variations between samples (*SI Appendix*, Fig. S3). Alpha and Epsilon samples were collected at the same time and from a population expected to have similar vaccination rates. We also analyzed data for Delta samples collected on two different days (*n* = 55 and 59) 23 d apart, and did not observe a significant batch effect.

The data presented here suggest that measuring “viral load” strictly by clinical cycle threshold (C_T_) value has limitations. It is well established that the “particle:pfu” ratio for viruses can vary between viral strains, cell type, and organism (11, 13). Our data indicate infectivity might vary between SARS-CoV-2 variants too. We propose that the RNA:FFU ratio should be investigated for future variants rather than relying on RNA C_T_ as the sole measurement of viral load. Our observation that Delta’s infectivity is increased compared to Alpha is in line with the previously observed increased transmission, spread, and likelihood of isolating live virus (8, 14). We observed similar infectivities for Delta and Epsilon, with trends toward increased infectivity and higher RNA levels for Delta samples compared to Epsilon. The advantage of both higher replication and infectivity might explain Delta overtaking Alpha worldwide; however, as Alpha replaced Epsilon, factors in addition to infectivity clearly play important roles in spread. Further work is needed to understand the molecular basis of this phenotype, including in vitro and in vivo studies. We chose to use VeroE6-TMRPSS2 cells as one of the most permissive cell lines for viral replication, which facilitate a physiologically relevant route of viral entry. Using a highly permissive cell line will help identify the largest number of infectious virions, without biasing measurements by using cells that have an intact interferon response. The increased infectivity in Delta clinical samples underscores the need for increased measures to prevent transmission to those who remain vulnerable, such as widespread vaccination, masking, distancing, and improved ventilation.

## Materials and Methods

### Viral Stability.

Variant stocks (*SI Appendix*, *Extended Methods*) were diluted 1:10, 1:100, and 1:10,000 and stored at 4 °C for the indicated period of time in the dark. Aliquots were removed each day and stored at −80 °C, and viral titers were measured by focus assay.

### Selection of Samples.

Clinical specimens identified as SARS-CoV-2 positive at the University of Washington Virology Laboratory were selected on March 25, August 3, and August 26, 2021 and stored in a monitored −80 °C until use (*SI Appendix*, Fig. S4). The use of deidentified positive specimens was declared exempt by the University of Washington Institutional Review Board (STUDY00010205). Specimens containing Alpha, Epsilon, and Delta variants were selected using RT–droplet digital PCR (*SI Appendix*, *Extended Methods*).

### RNA Extractions and RT-PCR.

Total nucleic acid in all clinical samples was extracted and amplified as previously described (12) using one of two sets of primers/probes: 1) WHO-E (15) or 2) Mills-sgE (12). C_T_ measurements of the AccuPlex SARS-CoV-2 Verification Panel (SeraCare, catalog #0505-0168) and of an in vitro–synthesized sgE transcript (12) were used to convert C_T_ to copy number in clinical specimens.

### Focus-Forming Assay.

SARS-CoV-2 viral titrations were conducted at the University of Vermont BSL-3 facility under an approved Institutional Biosafety protocol. Viral titer was determined by microfocus-forming assay using VeroE6-TMPRSS2 cells (Japanese Cancer Research Resources Bank #JCRB1819) (*SI Appendix*, *Extended Methods*).

### Statistical Analysis.

Data were analyzed with GraphPad Prism 8 and R (*SI Appendix*, *Extended Methods*).

## Supplementary Material

Supplementary File

## Data Availability

R code is available in GitHub at https://github.com/emilybrucelab. All other data are included in the manuscript and/or supporting information.
