# Supplementary material for: Measuring infectious SARS-CoV-2 in clinical samples reveals a higher viral titer:RNA ratio for Delta and Epsilon vs. Alpha variants
Source: Proc Natl Acad Sci U S A. 2022 Jan 20;119(5):e2116518119. doi: 10.1073/pnas.2116518119 (PMC8812544; doi:10.1073/pnas.2116518119)
Supplement: Supplementary File [file pnas.2116518119.sapp01.pdf]

PNAS
www.pnas.org

Supplementary Information for

Measuring infectious SARS-CoV-2 in clinical samples reveals higher viral titer:RNA ratio for Delta and Epsilon vs Alpha variants

Hannah W. Despres[1,#], Margaret G. Mills[2,#], David J. Shirley[3], Madaline M. Schmidt[1], Meei-Li Huang[2], Pavitra Roychoudhury[2,4], Keith R. Jerome[2,4], Alexander L. Greninger[2,4], Emily A. Bruce[1,*]

Emily A. Bruce
Email: emily.bruce@med.uvu.edu

This PDF file includes:

Supplemental Figures 1-4
Supplementary text
SI References

Other supplementary materials for this manuscript include the following:

Dataset S1

Supporting Information Figure 1

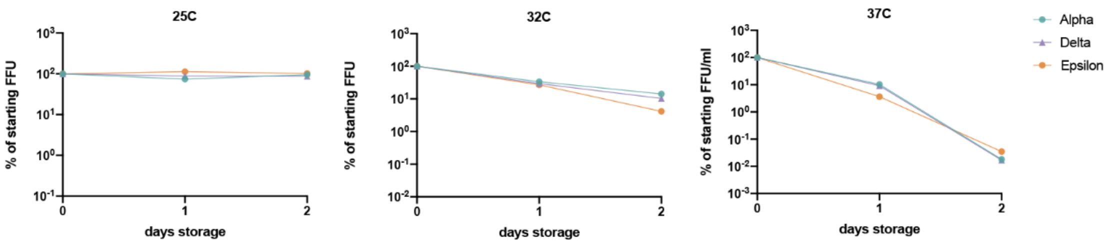

SI Fig 1. Effect of storage temperature on infectious titers. Stability of infectious virus from Alpha, Epsilon or Delta variants were measured by focus forming assay using viral stocks diluted 1:10, 1:100 or 1:10,000 in DMEM media and stored at 25°C, 32°C, or 37°C for the indicated times.

Supporting Information Figure 2

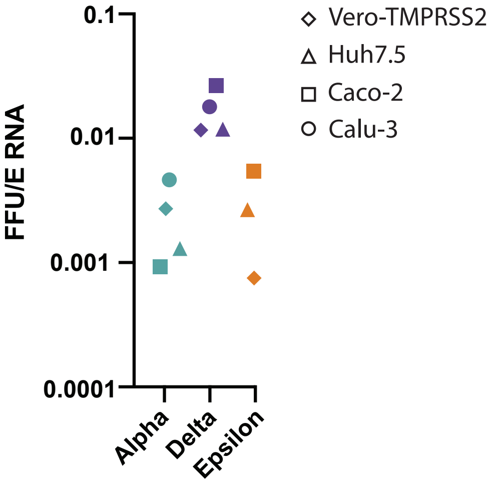

SI Fig 2. Variant Infectivity in Tissue Culture. Vero TMPRSS-2, Huh7.5, Caco-2 and Calu-3 cells were infected with stocks of Alpha, Delta and Epsilon viruses at an MOI of 0.1. Spernatants were collected and clarified at 24 hpi and the FFU and viral RNA levels (E gene RNA copies) were measured for each sample, in order to plot the infectivity for each variant.

Supporting Information Figure 3

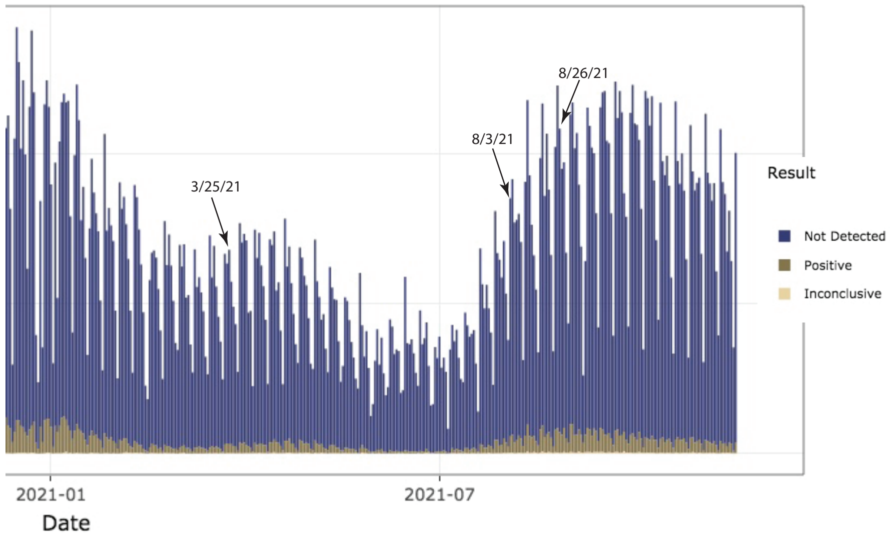

<u>SI Fig 3. SARS-CoV-2 Case Rates During Sample Collection Period.</u> SARS-CoV-2 testing statistics for the University of Washing Virology Laboratory around the period of sample collection are shown[1]. Research indicates that samples collected during periods of increasing cases tend to be collected closer to time of exposure than those collected during periods of decreasing cases[2]. All samples used in this study were collected during upswings in cases in the region.

Supporting Information Figure 4

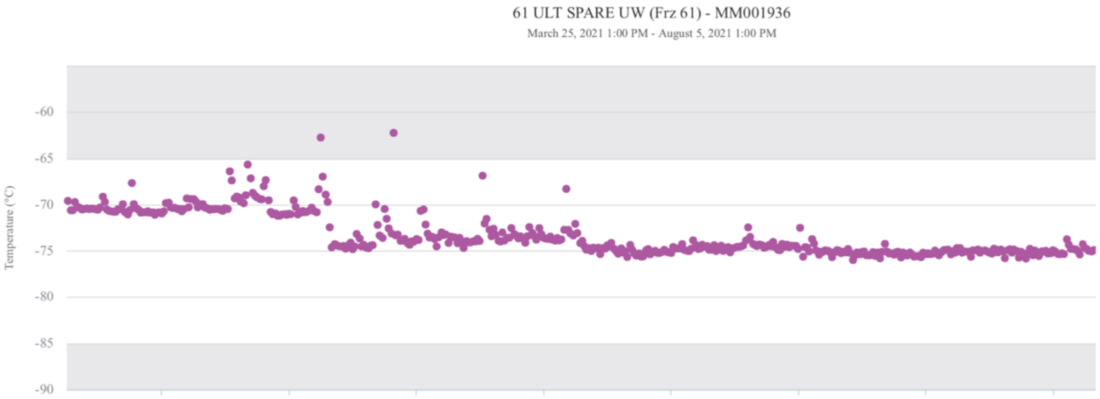

<u>SI Fig 4. Freezer Temperature During Sample Storage.</u> The samples for this study were stored in a -80C freezer in the clinical laboratory at the University of Washington Department of Laboratory Medicine & Pathology, Virology Division. The temperature monitor data for the freezer the samples were stored in is shown for the period of sample storage (March 25 to August 3).

Supplementary Information Text
Extended Methods

Generation of Viral Stocks
Clinical specimens positive for Alpha, Epsilon or Delta variants were used to isolate virus in Vero E6-TMPRSS2 cells, essentially as in[3]. Viral titer from successful isolates was determined by focus assay and this initial material was used to grow viral stocks by infecting Vero E6-TMPRSS2 cells at an MOI of 0.0005 at 32°C, overlaid with DMEM, 10% FBS, 1% HEPES and 1% Penn/Strep. Viral stocks were harvested when cytopathic effect was visible, and viral variant identification was confirmed by next generation sequencing.

Selection of samples
Clinical specimens were selected on three different days: Mar 25, Aug 3, and Aug 26, 2021. All specimens were nasal swabs in PBS collection kits (Greiner Bio-One cat. #456163) from drive-up community test sites in western and southern Washington. Aliquots from each sample were frozen at -80° C within 54 hours of sample collection (Mean ± SD: Alpha=40.1±10.4; Epsilon=41.4±9.9; Delta=39.3±10.2), and kept frozen until titering. Mutations in Spike indicating variants of concern (L452R and N501Y) were identified in clinical specimens using an RT-ddPCR assay similar to that we have previously described[4]. Specimens containing Alpha, Epsilon and Delta variants were selected by interpreting these results in combination with NGS surveillance done by UWVL in the period of each sample collection. In the week of the March collection, surveillance sequencing identified 31.5% Alpha (Y501), 29.5% Epsilon (R452), and 0.1% Delta; in the weeks of the August collection, samples were 0.4-0.6% Alpha, 0% Epsilon, and 98.1-98.5% Delta (R452) (https://depts.washington.edu/labmed/covid19/#sequencing-information).

Focus forming assay:
Vero E6-TMPRSS2, Huh7.5 or Caco-2 cells were infected, overlaid with 1.2% methylcellulose (Acros cat. #332620010) in DMEM and incubated for 24 h. As previous described[5], all dilutions were plated in duplicate with the exception of the neat clinical sample for which we only had sufficient material to plate one well per sample. All clinical specimens were titered in Vero E6-TMPRSS2 cells. Foci were detected with a cross-reactive rabbit anti-SARS-CoV N monoclonal antibody (Sinobiological, distributed by Thermo Fisher, Cat. #40143-R001; 1:20,000), a peroxidase-labelled goat anti-rabbit antibody (SeraCare, Cat. #5220-0336; 1:4,000) and peroxidase substrate (SeraCare, Cat. #5510-0030). Plates were imaged on a BioTek ImmunoSpot S6 MACRO Analyzer and foci were counted using an automated virus plaque counter as previously described[6] and manually corrected.

In vitro infectivity assay:
Vero TMPRSS-2, Huh7.5, Caco-2 or Calu-3 cells were infected with stocks of Alpha, Delta and Epsilon viruses at an MOI of 0.1. We collected clarified supernatants at 24 hpi and measured the FFU and viral RNA levels (E gene RNA copies) for each sample.

Statistical Analysis:
Data for Fig. 1A was analyzed with GraphPad Prism 8, using a linear regression analysis to compare the stability of the three variants to each other at each concentration, and determine if the slopes of the lines were significant from each other, or significantly different than 0. P-values for whether slopes were non-zero ranged from p=0.1 to 0.87. The slopes were not significantly different between variants; p=0.8, 0.1, 0.2 for high, medium and low concentrations of virus respectively. Data for Fig. 1B, Fig. 2A-C were analyzed using R[7]. For Fig 1B a linear regression model was fitted on log transformed titers consisting of an intercept, a main cell effect, a main variant effect and a variant-cell type interaction. The variant-cell type interaction coefficients for delta and epsilon (relative to an alpha reference) were not significant. For Fig 2 titers were log transformed, and FFU values below LoD values were set to one-tenth the LoD. Linear regression was employed to model the relationship between titer and C_T(E) or C_T(sgE). Interaction terms between the C_T values and variant were not significant and were dropped in favor of a simpler variant intercept only model, with all variants sharing a common slope.

SI References

1. UW Virology COVID-19 Dashboard. Accessed December 14, 2021. https://depts.washington.edu/labmed/covid19/#uw-virology-covid-19-dashboard

2. Hay JA, Kennedy-Shaffer L, Kanjilal NJ et al. Estimating epidemiologic dynamics from cross-sectional viral load distributions. *Science.* 2021; 373(6552).doi:10.1126/sceince.abh0635

3. Bruce EA, Mills MG, Sampoleo R, et al. Predicting Infectivity: Comparing Four PCR-Based Assays to Detect Culturable SARS-CoV-2 in Clinical Samples. EMBO Mol Med. 2021(e15290) doi: 10.15252/emmm.202115290

4. Perchetti GA, Zhu H, Mills MG, et al. Specific allelic discrimination of N501Y and other SARS-CoV-2 mutations by ddPCR detects B.1.1.7 lineage in Washington State. *J Med Virol*. 2021;93(10):5931-5941. doi:10.1002/jmv.27155

5. Graham NR, Whitaker AN, Strother CA, et al. Kinetics and Isotype Assessment of Antibodies Targeting the Spike Protein Receptor Binding Domain of SARS-CoV-2 In COVID-19 Patients as a Function of Age and Biological Sex. *MedRxiv [preprint].* 2020. doi:10.1101/2020.07.15.20154443

6. Katzelnick LC, Escoto AC, McElvany BD, et al. Viridot: An automated virus plaque (immunofocus) counter for the measurement of serological neutralizing responses with application to dengue virus. *PLoS Negl Trop Dis.* 2018;12(10):e0006862. doi:10.1371/journal.pntd.0006862

7. R: The R Project for Statistical Computing. Accessed September 2, 2021. https://www.r-project.org/
